# Interdisciplinary frontline teams in home-based healthcare services—paradoxes between organisational work structures and the trust model: a qualitative study

**DOI:** 10.1186/s12913-023-09695-y

**Published:** 2023-06-30

**Authors:** Ruth-Ellen Slåtsveen, Torunn Wibe, Liv Halvorsrud, Anne Lund

**Affiliations:** 1 Department of Rehabilitation Science and Health Technology, Faculty of Health Sciences, OsloMet- Oslo Metropolitan University, St. Olavs Plass, PO Box 4, Oslo, 0130 Norway; 2 Centre for Development of Institutional and Home Care Services in Oslo, PO Box 4716, Oslo, N- 0506 Norway; 3 Department of Nursing and Health Promotion, Faculty of Health Sciences, OsloMet- Oslo Metropolitan University, St. Olavs Plass, PO Box 4, Oslo, 0130 Norway; 4 Department of Rehabilitation Science and Health Technology- Occupational Therapy, Faculty of Health Sciences, OsloMet- Oslo Metropolitan University, St. Olavs Plass, PO Box 4, Oslo, 0130 Norway

**Keywords:** Primary health care, Health policies, Trust model, Interdisciplinary team, Frontline workers

## Abstract

**Background:**

Achieving access to quality healthcare services to ensure healthy lives and promote well-being for all at all ages is one of the United Nation’s Sustainable Developments Goals. In view of this goal, sustainable community healthcare services in Norway need to be urgently restructured in light of demographic changes, including an increase in the percentage of older adults in the country. National healthcare policies recommend finding new ways to organise and perform services using new technology, new methods and new solutions. The goal is to ensure greater continuity in the provision of services and softer transitions that enable service users to deal with a smaller number of people. The trust model is one such suggested organisational approach. The goal of the trust model is to involve service users and their next of kin in decisions that concern them while also trusting frontline workers’ professional judgement in assessing the need for services and adjusting them to address changes in the health of the users, thus making the services individually tailored and more flexible. This study aims to explore how organisational work structures influence the delivery of interdisciplinary home-based healthcare services.

**Methods:**

Observations, individual-, and focus groups interviews were conducted within community home-based healthcare services in a large Norwegian city with managers at different levels, nurses, occupational therapists, physiotherapists, purchaser-unit employees and other healthcare workers. Data was analysed thematically.

**Results:**

The results are presented in terms of themes— “Balancing on the margins: Negotiations between the time available, users’ needs, unforeseen events and administrative tasks” and “One gathered unit, but with different work structures”. The results identify organisational work structures that influence the performance of the trust model with regard to its intention of making flexible and individually tailored services available. However, these structures are different for the members of the interdisciplinary team, thus creating several paradoxes that need to be negotiated while fulfilling their daily responsibilities.

**Conclusion:**

This study suggests that it is crucial to pay attention to paradoxes and structures experienced by interdisciplinary frontline workers in home-based healthcare services, since they are unavoidable factors that need to be acknowledged when designing approaches for addressing the changes expected in community healthcare services.

## Background

Achieving access to quality healthcare services to ensure healthy lives and promote well-being for all at all ages is one of the United Nation’s Sustainable Developments Goals [1]. In view of this goal, sustainable community healthcare services in Norway need to be urgently restructured in light of demographic changes, including an increase in the percentage of older adults in the country [2]. Norway is well known for its service-based healthcare system, which is characterised by a primacy for formal care, a high level of public funding and robust local health and social care services infrastructure that are offered to and utilised by citizens from all socio-economic groups [3]. Moreover, Norwegian national healthcare policies outline the intention to assure support for older adults when they need it. The central question pertaining to this reform is, “What matters to you?”—be it encouragement in activity and participation or provision of assistance to cope with everyday life despite illness and loss of function [2, 4, 5]. In other words, the individual is given a greater opportunity to be involved and contribute to the content of the service provided. This reform further recommends finding new ways to organise and perform services using new technology, new methods and new solutions. The goal is to ensure greater continuity in the provision of services and softer transitions that enable service users to deal with a smaller number of people [2]. Consistent with their demographic developments, municipalities in Norway have selected many approaches towards addressing the shift expected to occur in community healthcare services. The trust model is one such suggested organisational approach. However, there are different ways of implementing this approach.

### The trust model as an alternative to the purchaser- provider model

Trust, within the model, is described as a strategy and work method that aims to create (i) services for the population based on their own choices and needs, thus addressing the need for more involvement from individual service users; (ii) more interaction between the community and the population, as well as between different levels within the community; and (iii) greater result orientation and implementation possibilities [6]. The goal of organising home-based healthcare services according to the trust model is to involve service users and their next of kin in making decisions that concern them, while also trusting frontline workers’ professional judgement in assessing the need for services and adjusting them according to changes in the users’ health, thus making the services individually tailored and more flexible [7]. This model, therefore, is an alternative to the purchaser-provider model that was implemented in Norway in the early 1990’s, as it encourages decision-making by smaller interdisciplinary frontline teams (IFLT). These teams might consist of occupational therapists, physiotherapists, nurses, purchaser-unit employees and other healthcare personnel. A key factor of the purchaser-provider model is the distinction between those responsible for the allocation of services and those who deliver them [8, 9]. This method of organising services involves reporting and controlling routines, which takes away focus from the service itself [7–9]. The purchaser-provider model introduced quality indicators for healthcare services to ensure users’ access to the services are legal and follow national guidelines. However, it has been criticised for reducing clinicians’ professional responsibility and discretion, which shifts attention from user involvement to institutional measurements and reports, thus making the services less sensitive to the particular needs of their users [9–12]. For many, this has led to a lack of personal continuity in availing services that have been fixed according to previous decisions and a lack of adjustment and continuous adaptation of decisions to suit service users [7, 10]. The trust model aims to address this challenge.

The trust model as described and implemented in Norway was found to be an area with limited research. Few relevant studies were identified, and no systematic reviews found. The Norwegian trust model is inspired by a similar model in Denmark which has been inspired by the Dutch Buurstzorg model focusing on providing home-based healthcare through self-managed teams of nurses [13–19]. However, these models are not transferable to the Norwegian trust model because of their limitation to one profession involved in the team [20]. The Norwegian model seems to be the only one focusing on interdisciplinary frontline teams. Despite this, some research from Norway shows similarities with the Danish research. Inhibitors mentioned in the literature are structural barriers, limited resources, lack of competences and motivation making it difficult to change the institutional patterns in an organisation, limited time for multidisciplinary meetings, decisions made outside the team and too many temporary workers [7, 13, 20, 21]. However, research also indicates positive outcomes as more interdisciplinary collaboration, increased flexibility in decision-making and increased personal continuity [7, 13, 20, 21]. Further, the studies showed that the effect of more responsibilities adjudged to the interdisciplinary frontline teams resulted in a reduction of the distance between the decisions of the users and the circumstances in which services were provided under the purchaser-provider model [7, 13, 20, 21]. At the same time, the interdisciplinary frontline team experienced the ability to perform flexible services, which they reported as motivating. However, Aspøy nuances this by discussing the focus on goals and results within the trust model, thereby claiming that this model should be considered a modification rather than a contrast to the purchaser-provider model [22].

In this study, a needs-led research process has been implemented, involving service users, their next-of-kin, managers, and interdisciplinary frontline workers in devising research questions related to the significance of the trust model in home-based healthcare services [23]. The needs-led research process shows, among other things, there is a need for in-depth knowledge related to the trust model’s intention to create more flexible and individually tailored services for service providers. Furthermore, the literature specifies the need to explore how the trust model affects service design and how its intention is “lived” by the organisational framework established by municipality healthcare service—issues that have not yet been adequately addressed by researchers or policymakers and need to be explored further [20]. Moreover, exploring the management of collaborative arrangements through various forms of meta-governance and how processes of collaboration may be facilitated or hampered by co-existing modes of governance have also been recommended [24]. In addition, Xyrichis and Lowton state that further research needs to be conducted at both the team and organisation levels to ensure that the improvement and maintenance of teamwork leads to improved quality in healthcare provision in the following decades [25].

This study investigates how the trust model is experienced by interdisciplinary frontline workers in home-based healthcare services. It aims to ameliorate our understanding by exploring how organisational work structures influence the delivery of interdisciplinary home-based healthcare services with the trust model as context. To the best of our knowledge there seems to be a research gap concerning facilitators and barriers related to interdisciplinary organisational models like the trust model in home-based healthcare. This study will address this knowledge gap.

## Methods

### Design

A qualitative study was conducted based on 32 observations of different meetings within the IFLT, and within the manager groups, 10 individual interviews and four focus group interviews carried out in Norway between March 2021 and April 2022 [26]. This involved multidisciplinary participation from the home-based healthcare services, community managers from different levels, nurses, occupational therapists, physiotherapists, and other healthcare professionals (see Table 1). They are hereby referred to as interdisciplinary frontline workers (IFLW).

**Table 1:** distribution of participants in focus group interviews (FG) and in-depth interviews (IDI)

Profession	FG Female	FG Male	IDI Female	IDI Male
Team manager	1		2	-
Manager assistant	1	1	1	-
Nurse	6	2	1	1
OT	3	1	1	-
Physiotherapist	1	3	1	1
Health care worker	1	1	-	-
Purchaser-unit employee	3	1	2	-
Total	16	9	8	2

The first author was continuously in dialogue with the managers in the two districts that participated in the study, striving to find time, and adapting the observations to the district’s daily routines. The managers chose the participants based on the work schedule and availability. The authors did not interact with the participants before the interviews or observations. Working within the home-based health service in the two districts that participated was the only inclusion criteria. We asked the managers to recruit IFLW that represented the professions and healthcare workers within the teams, and some team managers as well.

We were aware that the staff were very busy and therefore strived not spend too much time on interviews and observation. Moreover, this study was conducted during the pandemic which also created some limitations. Due to the lockdown and restrictions in Norway the observations and individual interviews were held digitally on zoom and other safe digital platforms provided by the healthcare services.

To maintain the anonymity of the participants, distinction between the different types of healthcare workers has been avoided. However, in some sections, differentiations between home nursing personnel and therapists have been made solely to highlight certain crucial points and differences between the two.

### Study setting

This study was conducted in the primary home-based care services provided in a large Norwegian community that has implemented the trust model. This municipality is divided into different districts, of which two participated in this study.

The trust model describes an organisational form of the home-based health care services that challenges to a reorganisation into smaller interdisciplinary frontline teams, with the purchaser-unit employee being an equal member of the smaller interdisciplinary teams, making decisions in continuous collaboration with the other team members and service users. This means to disband the purchaser-provider model, as well as the sectorial division of therapists, purchaser-unit employees and home healthcare workers that have been prominent in the community home-based healthcare. Instead, the model encourages to divide home-based healthcare services into several geographically organised interdisciplinary teams. Each team works independently and has its own manager. The IFLW within each team meet twice a week to discuss user cases and once a week to discuss their collaboration, expectations from work, workload, and other information that they perceive as important to share in an interdisciplinary environment. A geographical interdisciplinary team consists of a traditional home healthcare team, that is divided into two work teams,  a purchaser-unit employee, two occupational therapists and two physiotherapists, who are linked to both work teams.

### Data analysis

The data consisted of field notes from observations and transcriptions of the individual interviews and focus group interviews [27]. The first author was present during every observation and interview session and transcribed all the interviews, while the three other authors were alternately present on these occasions. All four authors maintained written observation notes from listening to what was discussed during the meetings. For validation, the observation notes were shared and discussed within the author group. Furthermore, reflexive thematic analysis inspired by Braun and Clarke was conducted to identify patterns of meaning across the data from the observations and interviews [28]. The epistemological stance adopted in this study is inspired by social constructivism, with the purpose of understanding the construction of the phenomenon within its social context [29]. The analysis was conducted by employing an inductive approach in which there was no attempt to fit the data into an existing theory, starting with familiarisation of the data and then moving to a systematic coding process before starting to explore, develop, review and refine themes [28]. Although only the first author wrote and performed the systematic coding and thematising by hand, all four authors contributed to this process through analysis meetings and workshops. Every meeting and discussion led to a deeper and more nuanced understanding of the identified patterns.

### Patient and public involvement

This study is part of a needs-led research endeavour [23]. We have included representatives of patients, their next of kin and service providers throughout the research process, including in the development of the interview questions.

## Results

The results demonstrate the differences in the understandings and practices related to the trust model experienced by IFLW, while also illustrating some examples of organisational challenges in negotiating the intention of the trust model.

The results are presented in terms of two overall themes— “Balancing on the margins: Negotiations between the time available, users’ needs, unforeseen events and administrative tasks” and “One gathered unit, but with different work structures”. The themes were developed through collective agreement and collaboration in the research team, and they continued to evolve during the writing process.

### Balancing on the margins: negotiations between time available, users’ needs, unforeseen events and administrative tasks

Resources, budgets, and unforeseen expenses, such as overtime work and the need to occasionally hire extra staff, are some of the issues that the participants in the study highlighted in different ways. The interdisciplinary frontline workers (IFLW) emphasised their concerns regarding having professional space for manoeuvring and confidence in their ability to adjust the time spent on the measures implemented based on their own assessments. Resources, such as the time spent interacting with the user and documenting the work conducted with and for the user, were mentioned as an important factor in making the necessary assessments to tailor services, focus on the user’s requirements and change measures according to the user’s needs. One IFLW commented:

It can take a while before you realise what the service-users’ needs are because you think or perceive that it also is a wish. In a way, it all has to do with time in these cases. There is also room to change it—if we have to increase time, then we will increase. All of it has to do with the service users’ needs.

At the same time, our results indicate that it is not always the user’s needs that govern the measures. Overarching guidelines, such as those related to finances and personnel, greatly affect the possibilities for manoeuvres in creating tailored services according to the user’s needs. Time frames that limit the possibilities for manoeuvres were a recurring theme in the interviews. Several IFLWs highlighted feeling a sense of pressure to deliver services within a given time frame, as well as limitations related to the availability of resources for providing the measures required by users. One IFLW expressed:

From an outsider’s perspective, the focus is to think mostly of the service-users’ needs and goals, but then part of the services is all about the economy where we are forced to reduce the estimated time. Even when the goal is to keep the patient at home, we must think about workforce, estimated time, downsizing… There is so much more we should have done, so much more we, on paper, should do and are instructed to do. But it is too costly in terms of overtime, and it is not possible to get everything done within our ordinary working hours. So, I feel that a lot of things are poorly undertaken due to time and staffing issues.

Furthermore, reporting in community nursing services and its effects on fulfilling daily responsibilities, having professional room for manoeuvres and adapting the services were also highlighted in the interviews. The BOOM report is a specific measurement tool that was especially underlined by the participants in the study, who defined it as follows:

A BOOM report is an overview of all hours spent on user-targeted work in a week per team. If there are large divergences in the actual hours against the forecasted hours, the deviation will be followed up and the reason for it has to be found. Therefore, you have estimated time and actual time—the time the manager has estimated that each person needs and what you have planned and actually done during the hours.

It was further informed that by spending less time on a responsibility than its estimated time fixed for the employees, they would be in danger of having this difference in time permanently reduced in their work lists if it lasted over time. If an IFLW spends more time than the estimated time indicated on the worklist on a certain activity, the IFLW needs to resolve this by trying to ensure a more even distribution of the time available. In such a case, if some users needed more time on their measures, others would receive reduced action time. Commenting on this issue, an IFLW claimed:

Trust management, having clear but few goals and giving employees more freedom to choose how they want to deliver assistance also helps the service users. So, it reflects an increased focus on what is important for you—for the user. But I don’t know if this is the reality. The idea was that we weren’t supposed to work according to time schedules but allocate our time to the service user who needed it. But the lists are full…. I honestly can’t see any difference in the time used; rather, there is an opposite effect—there is more to do. We are supposed not to use time on economy reports and look at BOOM reports, but we still do it. So, there is no difference there. My opinion is that you are still measured on a lot of factors.

This viewpoint was supported by another IFLW as well:

We are not allowed to increase the time dedicated to a user because it needs to agree with the BOOM report, so yeah, there is a lot of time pressure.

These examples indicate that these reports pose a hindrance to having professional room for manoeuvres to create flexible and adaptable services for users. It also appears that one often feels compelled to avoid reporting excessive changes for fear of losing action time. An IFLW commented:

The auxiliary workers said that one of the first thing they learn in their training is to cheat about the hours on their phone, or else the hours would be decreased. So, there is a lot of cheating that occurs by adjusting the time. You have the possibility to increase your time and do what you want with it, but it is then expected that you are honest the other way too… it has become an unwritten rule... but they must know that even if they cut their hours, we still don’t make better time because it just means that we refrain from hiring an auxiliary worker.

Apart from these issues, the participants talked about how the available time and resources limited their ability to cope with unforeseen events. Many of them also expressed how this affects opportunities for professional development, for working in interdisciplinary preventive fields and in interdisciplinary fields, which ultimately benefit users. An IFLW remarked:

You are always on the limit, and when you are always on the limit, you don’t have the capacity to handle unexpected things. It is certain that unexpected things will happen every day. Every day, there is something you haven’t anticipated or estimated, and it is certain that if you try to hire auxiliary workers, the answer will be, “No, it is not possible, we are over budget economically”. Then, there is also not much trust. When you address the fact that we have had to do a lot of overtime work, it is clear that it affects interdisciplinary measures as well as communication with the next of kin, the general practitioner and the hospital. In the end, we don’t get to plan the interdisciplinary work and we don’t get to move ahead. This has lasted for a long time. Still, no auxiliaries are hired…

The experience of failing to handle all given tasks, both foreseen and unforeseen, during a shift and the inability to offer flexibility and individual tailoring of services, as the trust model amplifies, was something that several IFLW emphasised numerous times.

### One gathered unit, but with different work structures

The results of this study indicate some variations in the working conditions of the different IFLWs. One of the prominent factors identified in both the observations and the interviews was that, while within the various professions in the interdisciplinary frontline teams had some common users, there were also many users who were not common to the different professions. For instance, the therapists estimated that about 50–60% of their user group had not received services from home nursing personnel, as a result of which they were not a part of the users that the professions had in common. The therapists further elaborated on this disparity by describing how they should not only prioritize among the users they have in common with the home nursing personnel but that these common users should also be prioritised among the large user group outside the team. In addition, it emerged that their room for manoeuvres in relation to priorities was also controlled by others. A manager revealed:

*“Now they (the therapist) are supposed to attend the nurses and other healthcare workers because we have allocated far too many long-term placements. This was addressed by the District Director, who said this doesn’t work. So, now you have to make more effort to work in an interdisciplinary manner—work in another way. Now physiotherapist, occupational therapist and purchaser-unit workers have to attend all meetings to work more preventively with interdisciplinary work…. And 60% of the service users didn’t have visits from nurses and other healthcare workers, meaning that other types of service users were given a lower priority since the service user who receives visits from nurses and other healthcare workers should be prioritized before others to decrease the allocation of long-term placements”.*

Another difference in the organisational structure of nurses and healthcare workers on the one hand and therapists on the other hand is that the former must continuously deal with the users’ changing needs and deal with the intake of new users of the service. They have no waiting list on which they can put the users, nor do they have any limit to the number of users they can have. If there is a new user or if the need for help increases suddenly among some users, it must be solved by the personnel who are at work, and priorities must be made on the measures and the allocated time for each visit to ensure that the work lists coincide with the personnel available. The therapists, in turn, manage their own working days, arrange their own home visits, and put all users on a waiting list, which, in turn, is controlled by a separate priority key. The therapists remarked:

Therapists don’t generate lists in the same way—we don’t work like that. Another difference is that they don’t have any waiting list. So, it is a little bit different…

Some observations mentioned that although there was a proposition for therapists to maintain work lists in the same way as nurse’s healthcare workers, this decision was not implemented due to disagreements on the issue. This was because if a therapist was sick, no one else would carry out the home visits scheduled for that day. They would be cancelled, and the therapist would have to make a new appointment when he/she got back at work again. In addition, therapists rarely have regular assignments depending on specific times of the day, such as medication, personal care and measurements. In contrast, nurses and healthcare workers have only regular assignments that cannot be cancelled. Hence, if personnel are ill, there arises a need for extra shifts or other personnel at work are expected to cover for their sick colleague.

Another factor mentioned by the participants in terms of the work lists was that since the nurses and healthcare workers had to deliver their assignments within a scheduled time, it affected their possibility to participate in interdisciplinary meetings or other such meetings conducted during a workday. In contrast, the therapists and purchaser-unit employees could schedule their days by themselves and adapt their tasks to attend meetings and other events. In addition, the same therapists and purchaser-unit employees were able to attend interdisciplinary meetings and collaborate with regular sectors within the healthcare unit, while the nurses and healthcare workers who participated in the meetings varied because of their work shifts. The staff stated the gravity of this challenge:

We do have interdisciplinary meetings, but not everyone who is in the meetings has something to do with the service users mentioned in the meetings. It is a shame that it is held only once a week…Since we have rotating shifts as nurses, we will maybe be able to attend every third meeting.

## Discussion

The aim of this study was to explore the influence of organisational work structures in the trust model on the delivery of home-based healthcare services by IFLW in a Norwegian city. The findings indicate a constant negotiation between delivering healthcare for the users and compromising policies and organisational structures, which can be related to Lipsky’s idea of the work structure [30]. The findings also demonstrate how frontline workers address users’ needs in different ways. The results discussed below draw on earlier research and national health policies while considering the trust model as the ideal structure and applying Lipsky’s street-level bureaucracy theory (30).

### The paradoxes of being a front-line worker in an interdisciplinary frontline team in street-level bureaucracy

This study demonstrates several paradoxes related to being an IFLW who needs to negotiate with their everyday work responsibilities to support users. Lipsky describes street-level workers, understood as the IFLW in this paper, as restrained by rules, regulations and directives issued by positions of authority, as well as by the norms and practices of their occupational groups [30]. Furthermore, he claims that the major dimensions of public policy with regard to the levels of benefits, categories of eligibility and the natures of rules, regulations and services are shaped by policy elites and political and administrative officials (30 p14). Although the IFLWs who participated in this study were expected to exercise considerable discretionary judgement in their field, as recommended by the trust model, they also had to negotiate the structural preconditions shaping their everyday work.

Supervision and control provide guidance for attaining bureaucratic goals—the clearer the goals and the better developed the performance measures are, the more finely tuned guidance can be [22]. However, a paradox arises when the IFLWs are provided with autonomy and are trusted to plan, formulate and decide the flexible and individually tailored services that should be provided to users. This is because they are simultaneously expected to address structural restrains regarding the time available to perform the services. The lack of personnel resources and the huge number of administrative tasks forced them to take recourse to shortcuts. The findings of this study indicate a prevalence of the practice of documenting and reporting dishonest estimations of the time used for providing services to users. This is often implemented as a way to achieve flexible and individually tailored services in the daily meetings conducted with the user and his/her varying daily routine despite structural restraints. There seems to be a constant need for negotiation between the users’ needs and what the designated time frame allows, which limits the professional “room for action” available for the IFLWs. It is evident that an organisation’s goals and results are considered of utmost importance, while trust is secondary [22]. The IFLWs also revealed having to negotiate between time and resources to cope with unforeseen events and achieve a feeling of doing a good and thorough job. Moreover, several participants emphasised the importance of availing opportunities for professional development and working preventively in interdisciplinary settings for the benefit of the service users. This may indicate a persistent feeling among the participants of not quite doing enough compared to what they feel obligated to do, want to do and ought to do. Instead, they are only able to work as “firefighters”—coping with urgent day-to-day tasks, which, in turn, might lead to dissatisfaction with their everyday work life.

Furthermore, the findings demonstrate that the trust models’ intention to establish smaller interdisciplinary frontline teams that are self-managed, possess sufficient professional space for action and empower employees has not been fully accomplished yet. It appears that, for many service providers, administrative reporting consumes to much of their time compared to their professional assessment of the service user’s daily needs. To cope with the burden of multiple responsibilities, IFLWs are driven to exercise discretion in processing substantial amounts of work with inadequate resources, thus compromising service users’ needs [30]. This means that they must develop shortcuts and simplifications, which creates conflicts between the client-centred goals held by the IFLWs and organisation-centred goals recognised by organisations and politicians (30 p18, 41). This, in turn, creates yet another paradox between the needs of the organisation to comply with the intention of the trust model and the daily work conducted with and for service users. Apart from this, a major difference among the IFLWs exists in terms of their reporting responsibilities and the extent to which they are “controlled”. Nurses and other healthcare staff work according to a fixed worklist that describes their tasks and the time available for serving every user. Moreover, they are bound to report the time spent at/with the users each day. In addition, they are expected to justify any difference between the planned user time and the actual time spent. In contrast, therapists and purchasers-unit employees set their own timetable and do not have any commands that need to be followed with regard to the task content or reporting the time spent at/with user each day. This indicates that the reporting system is split differently for different professions, notwithstanding whether they are co-organised as smaller interdisciplinary frontline worker teams or otherwise. It is, therefore, legitimate to inquire about how the different requirements and working conditions among IFLWs influence interdisciplinary activities and, in turn, the team’s ability to co-create flexible and individually tailored services.

Another paradox concerning IFLWs is the organisation’s right to enforce legitimate directives while maintaining illegitimate policy objectives (30 p18). Organisations emphasise the necessity for regulation and control through reporting measurements, fixed worklists, and close control regarding the use of extra personnel. However, the findings of this study indicate a feeling of being overcontrolled among the participants, along with a lack of trust and professional autonomy. It may be understood as distrust in policy objectives and the trust model’s intention. Another paradox that arises is in terms of control and trust between priorities based on the idealised dimensions of the trust model and the aim of creating tailored services. The trust model is supposed to enable the decentralisation of autonomy where IFLWs are trusted with the ability to make decisions based on their professional assessments. However, the findings indicate that a strong element of control persists. It seems that the overall aim within organisational structures tends to be difficult to achieve, as well as confusing and complicated to execute [30]. It is apparent that the purchaser-provider model continues to influence work structures, raising questions as to whether the trust model is an idealized goal that should be observed as a so-called trend and a vision rather than a work model that is actually implemented [31, 32]. Hence, this drives us to ask, as Håkansson does, whether there is a need to first reduce the focus on financial and structural control before attempting to reform the home-based healthcare sector through the promotion of trusting relationships [33].

### The paradox of being an interdisciplinary frontline team but having different work structures framing everyday work life

The aim of striving for interprofessional collaboration is, among other things, to achieve greater resource efficiency and improve the standards of care through a reduction in duplication and gaps in service provision, thus ensuring the delivery of flexible and individually tailored services, as well as better continuity of care [25]. Recent studies have pointed to the creation of team structures within teams to achieve better interprofessional collaboration [34]. However, our findings indicate that work structures within organisations and those based on professions also have a significant influence on framing the everyday work life of IFLT. There are several differences among the work structures of different IFLWs that need to be negotiated with the intention of well-functioning IFLT. This, in turn, impacts service providers’ abilities to provide flexible and individually tailored services.

One of the differences in work structures pertains to the disparities in the work hours of different IFLWs. The nurses and healthcare workers have fixed working hours and work shifts—day, evening, and night shifts. However, therapists work only day shifts and have the flexibility to start and end their workday a bit sooner or earlier if they wish to. Moreover, while nurses and healthcare workers must follow and complete fixed worklists based on the user’s needs for care during their shifts, therapists set their own daily schedules and decide both the time and the number of service users they shall attend to each day, as well as the amount of time they need to update their journals and carry out other administrative tasks. These are some of the differences that can certainly affect the dynamics among interdisciplinary frontline teams. The therapists are able to participate in meetings more regularly to a greater extent, while participation from nurses and healthcare workers is more varied—they often fail to attend meetings due to unforeseen events or time constraints. Such circumstances affect collaboration in various ways. Therefore, the question that arises is how interdisciplinary frontline teams can be effectively organised to work together in a more collaborative and better way, despite the differences in their working hours.

Another issue related to work structures that create a gap between the IFLWs within the teams is the difference between the nurses, healthcare workers and therapists when it comes to the allocation of new service users. The nurses and healthcare workers, to a greater extent, deliver the necessary health care and are, therefore, forced to accept all new service users and deliver services on the same day they are needed. In contrast, therapists are able to put service users on waiting lists that extend from one week to several months. In addition, IFLT nurses are required to send written referrals to the therapists when they observe the need for assessment from other professions within the IFLT. Clear team goals and a “holistic” approach towards service users are described as some of the advantages and aims of creating the IFLT [7, 25]. However, our findings indicate that interdisciplinary collaboration does not necessarily represent a continuum and the work structures that the IFLWs must negotiate with in their everyday work for natural reasons continue to perpetuate a distance between the professions, thus retaining a sense of “us and them” among the IFLWs.

The last work structure-related concern is the differences among IFLWs when it comes to selecting the service users to whom they should provide their services. The therapists estimated that approximately 50–60% of their service users were not in need of any other services. Meanwhile, several service users receiving care from nurses and healthcare workers do not require help from therapists. It is reasonable to assume that, since the IFLT does not have an entirely common user group, it becomes more difficult to establish close interdisciplinary collaboration while working on common goals. Although there is an acknowledgement that all users can benefit from an interdisciplinary approach in terms of receiving tailored services and the possibility for users to stay at home for longer periods, the issue of whether organisational work structures create limitations to the possibility of implementing such an approach should also be widely discussed.

It may appear that the responsibility for achieving the intention of the trust model and its implementation is pushed down on the IFLTs, who are left with the responsibility of addressing the challenges posed by organisational work structures to several areas and levels of goal achievement. This, in turn, might lead to these same workers being left with a bad conscience for failing to achieve the goals that have been set [34]. It might also relate to Håkansson’s study, which claims that managerial logic relates trust to the question of financial accountability, thus turning the former into a moral responsibility that involves acting in a certain way [33]. This seems to be further amplified by organisational structures that construct massively different worlds within one organization [34]. Therefore, it is appropriate to examine whether differences in the work structures that frame the IFLT create two different “worlds” within a team and, if so, how these two worlds can be bridged to create work structures that facilitate interdisciplinary work.

This study contributes to the ongoing discussion on possible ways to organise and deliver services in the years to come with the aim of meeting demographic changes, marked by an increase in the number of older adults. In addition, it contributes to the literature on challenges in making services individually tailored and more flexible. We suggest adopting a broader perspective on ways to organise home-based healthcare services to address the challenges noted in this study. Further research is necessary on issues such as interdisciplinary teams, co-creation of services and ensuring favourable performance of flexible and individually tailored services in practice among IFLWs organised according to the trust model.

### Strengths and limitations

This study design is based on three different qualitative approaches; observation, focus-groups, and in-depth interviews, complementing each other and adding strength to the findings. There is a diversity in the professional background of the interdisciplinary healthcare workers, which is a strength in representing the IFLT well. To collect qualitative data during a pandemic was challenging, both regarding access to the home-based healthcare services, but also regarding the restrictions that was in constant change and forcing us to have digital observations and in-depth interviews. However, the number of observations and interviews provided rich material and is a strength to the study. Even though some interactions, and personal contacts get lost in digital meetings, there were other benefits that we weren’t aware of before starting. When doing observations, it was much easier for the services to include us into their digital meetings (due to covid restrictions many of the meeting had to be digital), letting us be a natural part of it. And after we had presented ourselves, the aim of the observation and recorded with audio the participants consenting to the observation the authors turned off their video presentation and sound and was by that not a visible part of the meeting. This might have led to a safer environment for the participants and the authors could freely take notes and document in writing the conversation that took place in the meetings without affecting and disturbing the participants and meeting dialogue. It also strengthens the privacy and anonymity for the participants. In some of the meetings the participants were all participating from their individual computer, but in other meetings they were gathered in-person while the authors attended digitally from their individual computer (again this was related to the covid restrictions at that time). And in the latter meetings we were not able to see each participant that was in the room, and we have no information on the participants that attended the different meetings. Digital in-depth interviews are somewhat challenging because of the interaction that gets lost. However, it took shorter amount of time for the personnel to participate, being that they could do it from wherever they were.

The focus group interviews in this study however were done in person, and with people that worked together and therefore knew each other to some degree. It created a safe environment, and it was easier to make sure everyone was a part of the dialogue and was made room for to speak. The results presented are not necessarily transferrable outside its context but indicates some barriers and facilitators to be aware of and invites to further discussions.

## Conclusion

This study reveals that organisational work structures affect IFLWs in their delivery of interdisciplinary home-based healthcare services in several ways. Our findings highlight some of the paradoxes that exist between organisational work structures and the trust model, which the IFLWs must negotiate with in their daily work responsibilities to deliver flexible and individual tailored services for users. Moreover, the identified structures and paradoxes seem to have been insufficiently explored in terms of their organisation. Therefore, it is crucial to be aware of these paradoxes and structures. Since they cannot necessarily be avoided, they need to be properly acknowledged when designing approaches to address the shift that is set to occur in community healthcare services.

## Data Availability

The dataset derives from observations and interviews, all in Norwegian, and are not publicly available because of the terms of the data collection approval but parts of the data can be made available from the corresponding author upon reasonable request.

## Abbreviations

IFLT: Interdisciplinary frontline team
IFLW: Interdisciplinary frontline worker
